# Regional Management of Farmland Feeding Geese Using an Ecological Prioritization Tool

**DOI:** 10.1007/s13280-014-0515-x

**Published:** 2014-03-26

**Authors:** Jesper Madsen, Morten Bjerrum, Ingunn M. Tombre

**Affiliations:** 1 Department of Bioscience - Kalø, Aarhus University, Grenåvej 14, 8410 Rønde, Denmark; 2 Department of Bioscience, Aarhus University, Frederiksborgvej 399, 4000 Roskilde, Denmark; 3 Norwegian Institute for Nature Research, Arctic Ecology Department, The Fram Centre, 9296 Tromsö, Norway; 4 Present Address: Ramboll Oil & Gas, Environmental Assessment, Hannemanns Allé 53, 2300 Copenhagen S, Denmark

**Keywords:** Agricultural conflict, Compensation, Habitat selection, Refuge, Pink-footed goose, Stakeholder participation, Subsidy

## Abstract

Wild geese foraging on farmland cause increasing conflicts with agricultural interests, calling for a strategic approach to mitigation. In central Norway, conflicts between farmers and spring-staging pink-footed geese feeding on pastures have escalated. To alleviate the conflict, a scheme by which farmers are subsidized to allow geese to forage undisturbed was introduced. To guide allocation of subsidies, an ecological-based ranking of fields at a regional level was recommended and applied. Here we evaluate the scheme. On average, 40 % of subsidized fields were in the top 5 % of the ranking, and 80 % were within the top 20 %. Goose grazing pressure on subsidized pastures was 13 times higher compared to a stratified random selection of non-subsidized pastures, capturing 67 % of the pasture feeding geese despite that subsidized fields only comprised 13 % of the grassland area. Close dialogue between scientists and managers is regarded as a key to the success of the scheme.

## Introduction

During recent decades, population sizes of geese wintering in Europe have increased dramatically (Fox et al. 2010); they have expanded their breeding and non-breeding ranges (Madsen et al. 1999a) and in some cases dramatically changed their migratory schedules extending their stay in western Europe (e.g., Eichhorn et al. 2009). Due to loss of natural habitats and intensification of farming providing goose with energy rich and abundant food supplies, geese have converged to feeding on farmland from autumn through to spring (van Eerden et al. 1996; Fox et al. 2005). During the last 2–4 decades, more crop types which are sensitive to goose grazing have been introduced and are expanding in use at the expense of grasslands, such as winter cereals (e.g., Fox et al. 2005), and grass production has intensified with use of fertilizers and new grass types vulnerable to goose grazing (van Eerden et al. 1996). While foraging on waste crops as well as grass and winter cereals during dormancy in autumn and winter is generally unproblematic, conflicts with agricultural interests arise when geese feed on pastures and crops prior to harvesting, sprouting grass and winter cereals or new-sown cereals (van Roomen and Madsen 1992).

Alleviation of the conflict between geese and agriculture is mostly dealt with locally and on an ad hoc basis using scaring devices, sometimes coupled with accommodation areas for geese and compensation to farmers for lost crops (van Roomen and Madsen 1992; Cope et al. 2003; Tombre et al. 2013a). With the continued increases in goose numbers, their range expansions and resulting conflicts, the economic costs of managing the conflict have increased and European authorities have increasingly realized that more strategic approaches are needed to resolve the conflicts on a regional basis (Scotland: Cope et al. 2005; Crabtree et al. 2010; The Netherlands: Kwak et al. 2008; the International Wadden Sea: Madsen 2010). Cost-benefit analyses have shown that schemes providing geese with accommodation areas give value for money from an overall societal perspective (Vickery et al. 1994a; MacMillan et al. 2004). Cost-efficiency will increase with an optimization of an ecologically based design of schemes in terms of size and distribution of accommodation areas (Amano et al. 2007; Jensen et al. 2008) and management of food quality and/or quantity in fields (Vickery et al. 1994b; Vickery and Gill 1999; McKay et al. 2001; Si et al. 2011) to accommodate most geese per unit area. However, although the ecological knowledge and advice have been widely applied in the management of the goose-agricultural conflict, the evaluation of the effectiveness of schemes is generally lacking.

During spring, pink-footed geese (*Anser brachyrhynchus*) from the Svalbard-breeding population congregate in the county of Nord-Trøndelag, central Norway, before their onward migration to stopover sites in Vesterålen, north Norway and the breeding grounds. During their stay in Nord-Trøndelag, the geese feed intensively on sprouting pastures and newly sown cereal fields and this causes a direct conflict of interests with the farmers (Søreng 2008; Bjerke et al. 2013). In response, farmers have been using different means of scaring the geese away. However, increasing complaints by farmers in the early 2000s led to the introduction of a subsidy system from 2006 onward by which farmers can be subsidized to accommodate the geese (Tombre et al. 2013b). There has, however, not been sufficient funding to subsidize all farmers and the regional authorities had no systematic tools at hand to support a prioritization for the allocation of the funds. In the first three years, the subsidies were distributed ad hoc according to requests from farmers and some overall evaluation by the responsible agronomic managers.

To support the prioritization of which fields were most susceptible to goose grazing, we developed a system of ranking all fields in the region according to their suitability to pink-footed geese, based on a statistical spatially explicit model and knowledge of previous goose use of the fields (Jensen et al. 2008). Basically, the model predicted that the most suitable fields were relatively large and positioned close to the coast or lakes where geese roost. The prioritization ranked each individual field based on the rank sum its size, connectivity to other fields, years of historic use by geese, and proximity to roost. Since 2009, the regional authorities have used this prioritization as their main tool to distribute the subsidies to farmers.

In this paper we evaluate the resulting efficiency in terms of how subsidies were actually used. We briefly describe the process toward the implementation of research results in local management. To our knowledge, this is one of the first attempts to evaluate the ecological cost-effectiveness of a goose-agriculture management system at a regional level.

## Materials and Methods

### Study Area

**Fig. 1:**
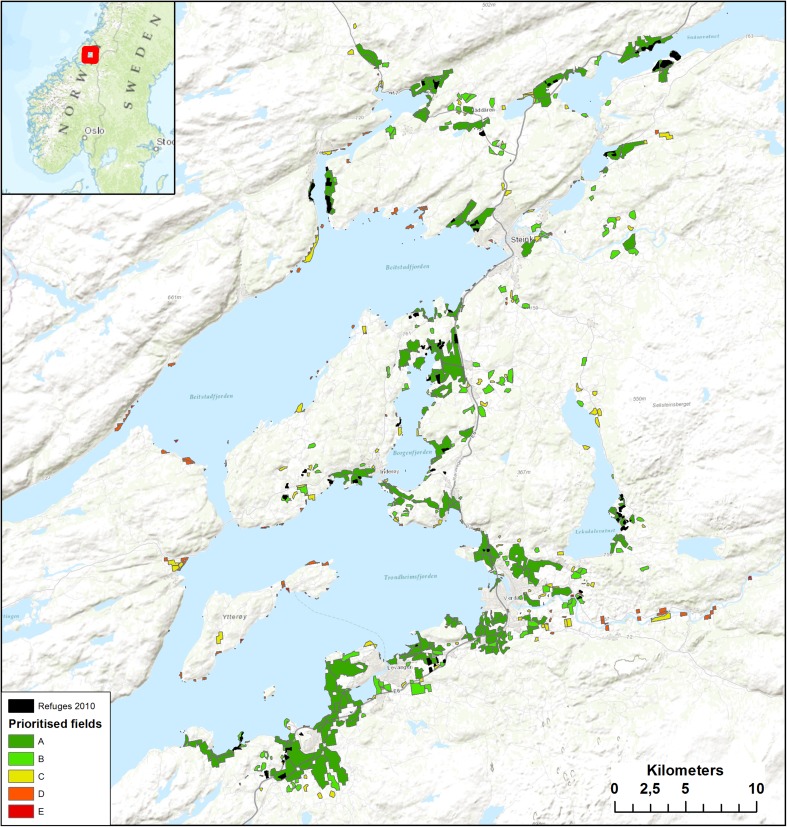
Study area in Nord-Trøndelag, the Trondheimsfjord area, showing the subsidized areas in 2010 (*black*) on top of the prioritized areas, ranked in 20 % fractiles (*A* is highest 20 % priority fields, *E* is lowest priority). *Inset map* shows the position of the study area (*red*) in central Norway

The geese make a stopover in the lowlands of the interior part of the Trondheimsfjord in the county of Nord-Trøndelag, central Norway (Fig. 1). The landscape consists of a mixture of farmland and woodland; farmland crops consist of spring-sown cereals, pastures and potatoes. Most harvested cereal fields are plowed in the autumn, but some stubble fields will remain until the subsequent spring when they are plowed and sown during April–May.

Pink-footed geese from the Svalbard-breeding population, which winters in Denmark, The Netherlands, and Belgium, make a stopover in the region during April and May. The population as a whole has increased from around 30 000 in the 1980s to 69 000 in 2010 when the field study was carried out and reaching an unprecedented peak of 81 500 in 2012 (Madsen and Williams 2012; Madsen et al. 2013). Flocks of geese started to use the Trondheimsfjord area in the late 1980s, but since then, the entire population calls in for more 2–4 weeks before onward migration to stopover sites in Vesterålen, north Norway and ultimately, the Svalbard-breeding grounds (Madsen et al. 1999b; Tombre et al. 2008). In 2010, when field work was carried out, the first flocks of pink-footed geese arrived in the first days of April and around 17 May most geese had left the region. On 3 days, when geese were counted by a team of observers throughout the region, a total of 44 254 (24 April), 51 739 (2 May), and 61 256 (8 May) pink-footed geese were registered (Bjerrum et al. 2011).

**Fig. 2:**
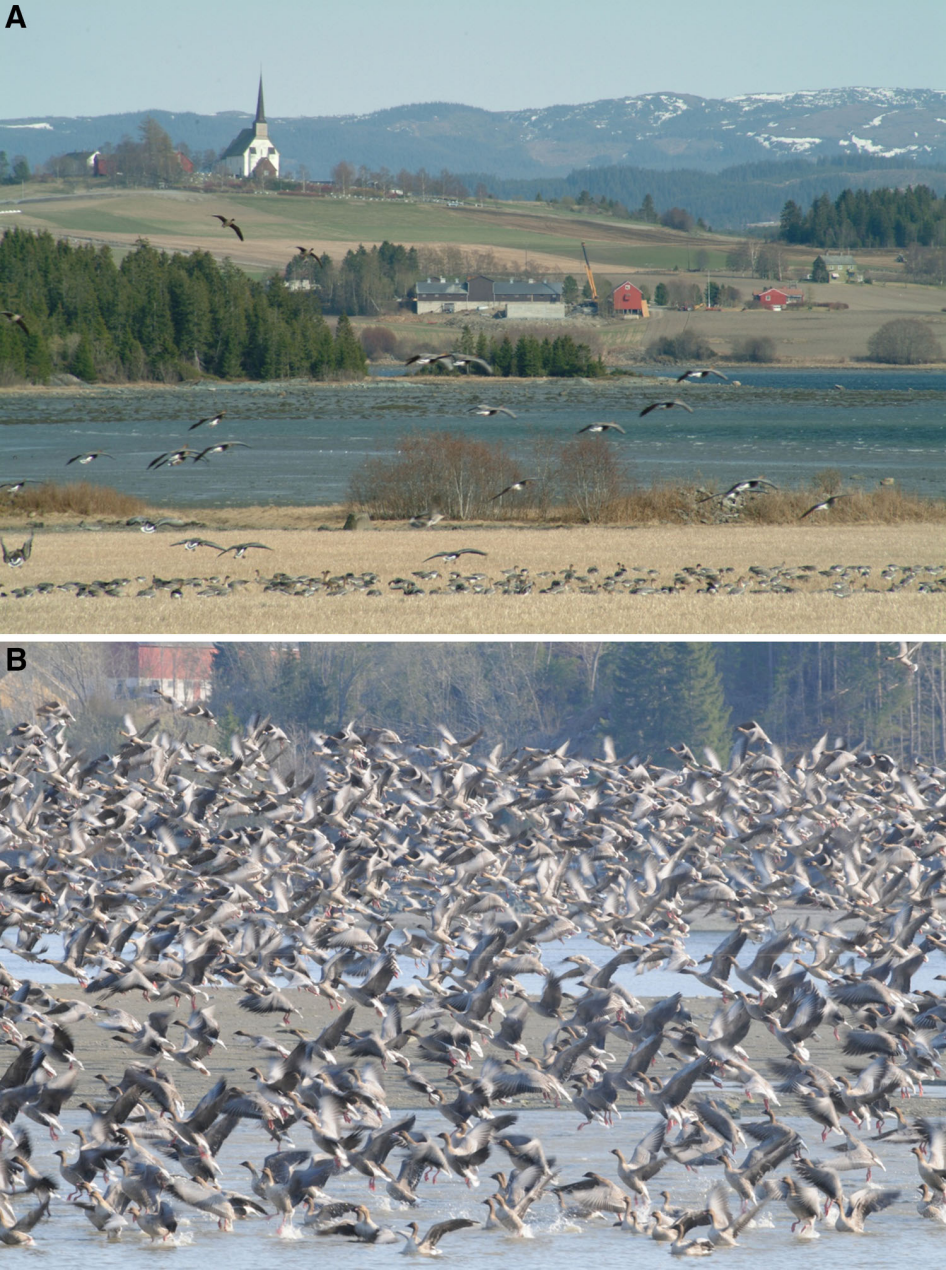
Pink-footed geese rely on foraging in a cultural and intensively farmed landscape in mid Norway (**a**). They roost on the nearby coasts, lakes and rivers where they aggregate at night and during the middle of the day to rest. They often leave the roosts in big flocks to fly to the foraging fields (**b**). *Photos* Per Ivar Nicolaisen

On arrival in the Trondheimsfjord area, geese feed on grass in pastures and waste grain in stubble fields. They roost on the nearby coasts, lakes, or rivers (Fig. 2). Once sowing of spring cereal fields commences during late April to mid-May, geese also forage on the newly sown grain (Madsen et al. 1999b). In 2010, little snow and an unusually cold winter, combined with a humid and cold May, deferred the onset of spring. However, the phenology of the goose migration did not deviate from normal springs but it had the effect that the geese were primarily grazing on grass and stubble fields while foraging on newly sown fields was negligible due to delayed sowing.

In 2010, a total of 1.44 million Norwegian kroner (195 000 EURO) was assigned by the national and regional agricultural authorities for subsidizing farmers in Nord-Trøndelag to allow geese feeding on their grasslands and newly sown fields. Rate of subsidy was 3000 kroner per hectare for pastures and 1000 kroner per hectare for newly sown cereal fields. The rates for pastures are higher because this is where the highest grazing pressure is exerted and because in some years the sowing of grain takes place so late that the majority of geese have departed. The amount of money made available has been a political compromise and not based on a damage assessment; the rates of subsidy have been based on a qualitative assessment made by the agricultural authorities. As more farmers applied to join the scheme than were available funds a prioritization of fields had to be done. The prioritization primarily followed the ranking of fields based on the suitability of fields for geese and previous goose use proposed by Jensen et al. (2008), and the authorities also prioritized to adjoin fields to create larger units of subsidized areas (hereafter referred to as refuges).

### Methods

From the regional authorities (Agricultural Department, County of Nord-Trøndelag) we obtained GIS layers with the appointed refuges for 2009 and 2010. We compared the position of refuges according to the ranking of suitability of fields that we had previously made on the basis of goose distribution data collected during 2004–2007 (Jensen et al. 2008).

To assess the effect of refuges we compared the density of goose droppings inside the refuges with the density on non-refuge areas in 2010. The non-refuge areas were randomly selected from the pool of all fields within 2 km from refuges (the 2 km radius was chosen to have sufficient fields to randomly select from), the fields being weighted by similarity in size/circumference to the refuge areas. During 10–12 May 2010, four field teams drove around to the selected fields and counted droppings in the majority of refuges and appointed random non-refuges. In total, we counted densities of goose droppings on 228 fields, of which 90 were refuges (out of a total of 106) and 138 were non-refuges. In each field we counted goose droppings in three circles, each with a radius of 2 m. The first circle was placed in the center of the field, the second at two-thirds of the distance from the edge to the center, and the third at one-third of the distance to the edge. The mean of the counts in the three circles was considered an estimate of the overall goose grazing pressure in the field because geese were expected to exert maximum use of the field centers and declining use toward the edges due to presence of roads, woodlands or constructions. Since goose droppings are intact and visible for 3–4 weeks depending on the intensity of rain (Madsen 1985a), the dropping density gives an expression of the accumulated use of a given field for most of the relevant season. Only pink-footed geese occurred in the fields during spring. We assume that there was no major difference in the quality and quantity of forage in the fields which were visited. All pastures were fertilized with manure in the course of May and fields were mainly used for hay-cutting (in June/July). We know of no farmers who have managed their grasslands to optimize for geese or the opposite.

Because of the delayed sowing in 2010, we obtained too little data for new-sown fields for analysis. Therefore, we put emphasis on the goose use of refuge versus non-refuge pastures but we also include stubble fields because some stubble fields were subsidized as they were to be turned into new-sown fields potentially subject to goose damage (which they were not to any significant degree in 2010). Geese defecate more often while foraging on grass compared to grain (Madsen 1985b); hence we did not compare the density of droppings across crop types.

As the goose grazing pressure on the fields can potentially be influenced by scaring measures on neighboring non-subsidized fields, we tried to obtain information about local scaring activities. We only succeeded in getting information for 46 % of the fields, of which only 3 % were positively known to have some intensity of scaring (mostly farmers scare geese away by walking or driving into the fields). Therefore, there is not enough data to evaluate the effect of scaring in this study.

To see if our observations were in accordance with the original model predictions, we analyzed the relationship between dropping densities, refuge size and refuge distance to nearest coast or lake (used as roosts). In cases where refuge fields adjoined, we used the grouped field size as the refuge area and the average dropping density of adjoined fields. Data on dropping densities and refuge distance were not normal or log-normal distributed (Shapiro–Wilk test, *p* < 0.001). Therefore, we applied non-parametric Spearman’s correlation analyses (carried out in R 2.15.2^©^; R Development Core Team 2012).

## Results

### Size and Quality of Refuges

In 2010, the total refuge area in Nord-Trøndelag was 402.4 ha of which 90 % was pasture. The mean size of the refuge fields was 3.80 ha (*n* = 106; SD = 3.14; range 0.20–14.41). Out of the 106 refuge fields, 48 were adjoined to other refuges. Including the grouped areas, the resulting average refuge size was 5.63 ha (*n* = 78; SD = 4.67; range 0.20–25.57).

**Fig. 3:**
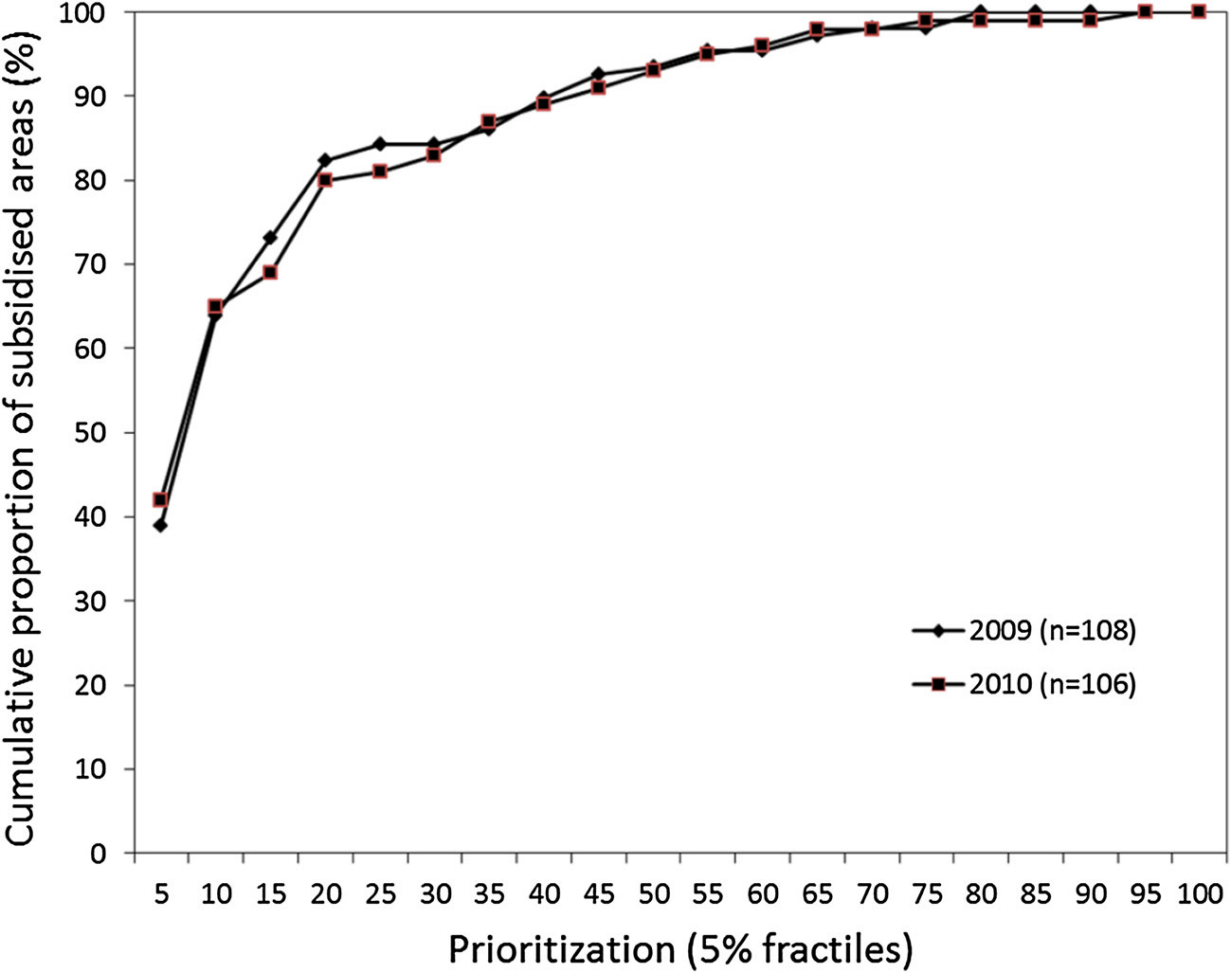
Cumulative distribution of refuge areas in Nord-Trøndelag, 2009 and 2010, compared to the prioritization by Jensen et al. (2008). Prioritized fields are ranked in 5 % fractiles according to their predicted suitability (lowest fractiles have the highest priority)

Comparing the spatial distribution of refuges in 2010 with the prioritization based on the data from 2004 to 2007, it is seen that the refuges were primarily selected among the highly prioritized areas (Fig. 1). Further, both 2009 and 2010, there was a good accordance between the fields selected as refuges and the prioritization, grouped into 5 % fractiles of the total pool of fields in the region, viz. a total of 1008 fields in four municipalities (Fig. 3). Hence, on average between the years, 40 % of the refuges were distributed in the top 5 % of the highest ranked fields and 80 % of refuges were with the top 20 % of the ranking (Fig. 3).

### Goose Use of Refuges

**Fig. 4:**
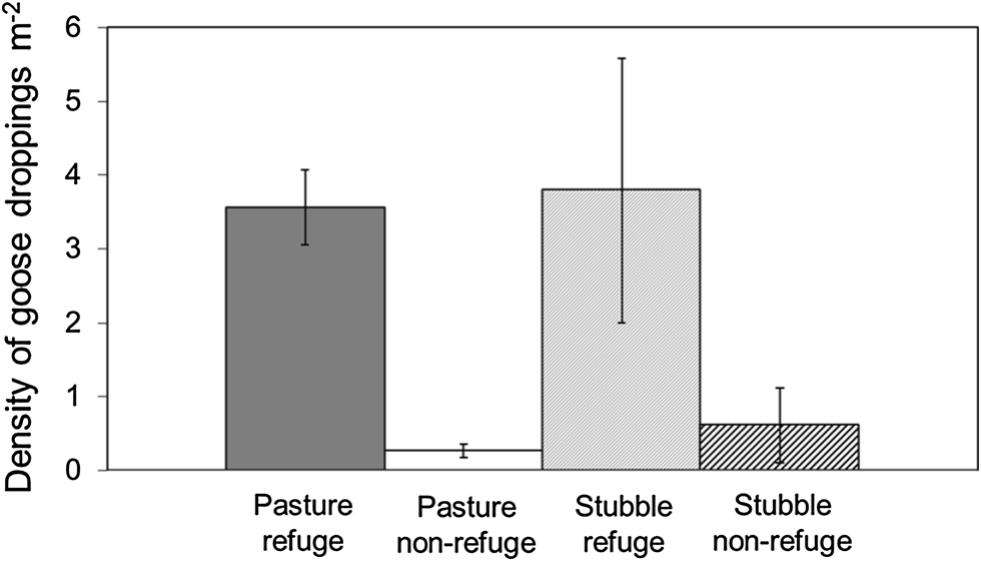
Mean density of goose droppings (±SE) as a measure of goose grazing pressure during spring 2010 on pastures and stubble fields with and without refuge status, respectively

In 2010, the density of goose droppings was significantly higher on refuge fields than on non-refuge fields, on both grass and stubble (Student’s *t* test (two-sided), grass: *t* = 7.29, df = 193, *p* < 0.0001; stubble: *t* = 2.18, df = 12, *p* = 0.025) (Fig. 4). The dropping density was significant negatively correlated with refuge distance to nearest coast/lake (*r*_s_ = −0.54, *p* < 0.001, *n* = 78). There was no significant correlation found between dropping density and refuge size (*r*_s_ = 0.19, *p* = 0.14, *n* = 78).

### Estimation of Total Refuge Use

Based on field data collected in 2010, we can roughly estimate the proportional share of the total goose grazing pressure on pastures captured by the refuges. According to the species distribution model for pink-footed geese in Nord-Trøndelag (Jensen et al. 2008), the total suitable area for geese comprises 104 km^2^. Further, on basis of field data collected during 2008–2010, we know that the proportion of grassland is rather constant at approximately 26 % of total farmland in Nord-Trøndelag each year (J. Madsen unpubl. data), i.e., covering c. 27 km^2^. Refuges with pastures comprised 3.6 km^2^ which compares to a suitable non-refuge pasture area of 23.4 km^2^. With the observed average density of goose droppings on refuges and non-refuges of 3.56 and 0.27 droppings m^−2^, respectively, we can multiply the respective grassland areas with dropping densities to derive the total grazing pressures. We calculate that 67.1 % of the goose grazing pressure on grassland in Nord-Trøndelag is exerted on refuges, despite that they only comprise 13.4 % of the total pasture area available.

## Discussion

Management of the conflict between geese and agricultural interests is complex and dynamic, comprised of a mix of behavior (human and wildlife), ecology, socio-economics, politics, and geography (Nyhus et al. 2005). Accordingly, the combination of increasing numbers of geese which rely on foraging on farmland in Europe has led to an increasing political pressure by farming interests calling for more economic compensation (notably in The Netherlands, Germany, Scotland, Norway, Sweden, Bulgaria, Denmark but conflicts are more widespread). The conflict situation differs between countries in terms of the ecological and agricultural settings, available regulatory instruments, history of goose conflicts (including human perception of geese in general), and political willingness to pay compensation for damage or for the accommodation of geese. It is obvious that in such a spiraling situation a strategic and adaptive management approach is advantageous, including stakeholder involvement (Berkes et al. 2003; Armitage et al. 2009).

The Nord-Trøndelag case is an example of a gradually evolving strategic management scheme operating at a regional level. The subsidy scheme was implemented following a more than ten year long political discussion in Norway, starting with increasing conflicts at goose staging areas in north Norway (Eythórsson 2004; Tombre et al. 2013a), moving on to Nord-Trøndelag (Søreng 2008). Originally, the national authorities were reluctant enter a scheme because of the possible precedence and the fear for increasing economic demands but eventually a so-called “environmental subsidy scheme” was agreed and initiated as a pilot project in 2004 and fully implemented in 2006, co-funded by the agricultural and environmental ministries. Due to limited funds and high request for subsidies from farmers, the regional authorities in north Norway as well as Nord-Trøndelag soon asked for ecological evidence-based guidance on how to distribute the subsidies to get most value for money in terms of accommodating most geese. In Vesterålen in north Norway, geese (pink-footed geese and barnacle geese) feed on a limited number of pastures in a narrow zone between the coast and the mountains, hence it has been relatively easy to prioritize fields based on regular counts of geese (Tombre et al. 2013a, unpubl. data). In Nord-Trøndelag, the geese are distributed much more widely over a mosaic landscape which has required a statistical predictive approach to prioritization.

The analysis shows that the regional and local authorities have to a very high degree followed the recommendations based on the prioritization. Refuges were primarily positioned close to roosts and fields were merged to create larger units, which was probably the reason why our data did not show a relationship between refuge size and goose densities. The field data from 2010 confirm that the use of the prioritization resulted in a high efficiency, accommodating around 67 % of the total regional goose grazing exerted on pastures on 13.4 % of the available pasture area. Furthermore, outside the subsidized fields, the grazing pressure was generally so low that it is unlikely that the geese cause damage to the yield of grass (Bjerke et al. 2013).

The calculation of refuge efficiency is sensitive to the estimate of the total area used by the geese, which is likely to expand with increasing goose numbers due to density-dependent effects; however, even with a 50 % increase in the area, the refuges would still capture more than 50 % of the geese. Further, because geese prefer fields close to the roosts, as confirmed by the results of statistical analyses from 2010, goose grazing pressure is generally expected to remain relatively low in the newly colonized areas furthest away from the roost sites. However, the existing model cannot account for the situation that geese start exploiting new sites outside the present range, and this calls for a regular update of the model, based on new empirical information of roost sites occupied by the geese.

We also showed that refuges on stubble/new-sown fields had a positive effect; however, the need for refuges on new-sown fields varies vastly between years. The spring of 2010 was rather late and the geese only used the new-sown fields at the very end of their stay; in early years, the grazing pressure and hence, potential damage to new-sown fields will increase.

In the present study we cannot account for the effect of scaring in the non-refuge areas which may have an additional positive effect on goose use of refuges because birds are moved there (see for example Tombre et al. 2005). However, the capacity of grassland, i.e., biomass and primary productivity within the period geese are there, will ultimately set a limit to the density of geese that can be accommodated on the refuges. To advance the prioritization and efficiency of the scheme further we need to understand better the dynamics of how geese make decisions about their daily choice of fields. Baveco et al. (2011) have provided a first step to make a model for goose management at the scale of The Netherlands, but outside the plant growing season. To make a realistic evaluation for Nord-Trøndelag, it is be a challenge to include the goose population development, dynamics of crops, vegetation growth and effects of scaring.

As long as the farmers join the scheme it may be argued that the scheme is mitigating economic costs from the farmers’ perspective. It should be noted, however, that many farmers still consider the scheme unsatisfactory, either because the subsidy rate is regarded too low or because those who are not involved in the scheme may spend a significant amount of time protecting their fields by chasing the geese off their properties (Søreng 2008). Therefore, to ensure the sustainability of the scheme, it will also be important to incorporate estimates of economic costs of various scenarios of subsidies versus scaring as well as the wider societal benefits of the scheme, for example the positive values of people observing geese (sensu MacMillan et al. 2004).

Since we are dealing with a migratory species, management actions taken at one stopover site along the migratory pathway may affect the migration strategy of the entire population (Klaassen et al. 2006). Therefore, it is valuable to take a flyway perspective to optimize future management strategies, as costs of management can vary between sites (Klaassen et al. 2008), depending on goose migration schedules in relation to availability of crop types, their timing of growth and management actions taken. The Svalbard pink-footed goose has been selected as the first European case for international adaptive management of a migratory species under the African-Eurasian Waterbird Agreement (Madsen and Williams 2012), and the longer-term alleviation of the spring agricultural conflict is one of the themes that will be addressed. Accordingly, flexible flyway-based management tools and actions which are recurrently monitored, evaluated and adjusted are required.

In an analysis of the political–sociological conflict situation in Nord-Trøndelag, managers and farmers’ organizations expressed that a key to the success of the scheme was the close co-operation between scientists and managers in developing and tuning the subsidy scheme (Søreng 2008). The development of a spatial model and its planned use as a tool for prioritization of fields for subsidizing farmers resulted from a research project (starting January 2005) and the County Governor was closely involved in the process. This ensured that user needs (such as user friendliness) were taken into account in the model and that scientists got a higher degree of legitimacy as partners in the management process and when meeting farmers in the field. The communication between scientists, managers, farmers and other stakeholders was brought about by several meetings. Hence, in October 2007, a regional conference in Nord-Trøndelag focusing on the conflicts between geese and agricultural interests was organized by the County Governor. Relevant environmental and agricultural authorities as well as farmers’ associations and conservationists were invited. Here the model, its application and outputs were presented (see Jensen et al. 2008). As a follow-up, we were invited to participate in a consultation meeting organized by the County Governor in the autumn of 2008. Here, representatives from the involved municipalities were also attending, and a detailed description of how the model was built and how it could be used was elaborated further. The managers agreed that the model would be a useful tool and that it would be implemented in the future distribution of subsidies. One challenge was to merge the model output with maps of landownership in order to identify fields at the priority list. As a result, a GIS database was produced, held at the County Governor (www.gint.no), showing all refuge areas from the priority list and the areas that were subsidized based on the available funding. This has formed the basis for prioritization by the County Governor and municipalities since 2009. The publicly available GIS has given landowners the opportunity to follow the allocation of subsidies. Managers have used the tool to justify the allocation of subsidies, specifically in cases where landowners have complained about not having received support. Based on annual monitoring of goose use of the region as well as new research results (see www.nina.no for annual reports) we have had annual consultations with the County Governor to discuss how development in goose use of Nord-Trøndelag might affect the priority list (see also Tombre et al. 2013b). So far it has been agreed to stay with the original listing.

## Conclusions

To alleviate the conflict between spring-staging geese and agricultural interests, authorities in Nord-Trøndelag, Norway have effectively allocated subsidies according to an ecologically based tool to prioritize fields most suitable for accommodating geese. This strategic approach has increased the cost-efficiency in terms of goose numbers accommodated under the scheme. Close dialogue between scientists and managers is judged as one of the keys to the success of the current scheme and its development. The continued successful application of the prioritization tool will require adjustments as the goose population continues to grow and to account for an adaptive dual strategy for attracting geese to certain fields while scaring them away from fields where they are not wanted. It is also recommended to take a wider societal as well as international, flyway-based perspective on the regional conflict.
